# A Novel Dual Bacteria-Imprinted Polymer Sensor for Highly Selective and Rapid Detection of Pathogenic Bacteria

**DOI:** 10.3390/bios13090868

**Published:** 2023-09-03

**Authors:** Xiaoli Xu, Xiaohui Lin, Lingling Wang, Yixin Ma, Tao Sun, Xiaojun Bian

**Affiliations:** 1 College of Food Science and Technology, Shanghai Ocean University, Shanghai 201306, China; 2 Laboratory of Quality and Safety Risk Assessment for Aquatic Product on Storage and Preservation (Shanghai), Ministry of Agriculture, Shanghai 201306, China; 3 Shanghai Engineering Research Center of Aquatic-Product Processing & Preservation, Shanghai 201306, China

**Keywords:** dual bacteria-imprinted polymer, electrochemical sensor, bacterial detection, molecularly imprinted polymer, *o*-phenylenediamine, *Escherichia coli* O157:H7, *Staphylococcus aureus*

## Abstract

The rapid, sensitive, and selective detection of pathogenic bacteria is of utmost importance in ensuring food safety and preventing the spread of infectious diseases. Here, we present a novel, reusable, and cost-effective impedimetric sensor based on a dual bacteria-imprinted polymer (DBIP) for the specific detection of *Escherichia coli* O157:H7 and *Staphylococcus aureus*. The DBIP sensor stands out with its remarkably short fabrication time of just 20 min, achieved through the efficient electro-polymerization of o-phenylenediamine monomer in the presence of dual bacterial templates, followed by in-situ template removal. The key structural feature of the DBIP sensor lies in the cavity-free imprinting sites, indicative of a thin layer of bacterial surface imprinting. This facilitates rapid rebinding of the target bacteria within a mere 15 min, while the sensing interface regenerates in just 10 min, enhancing the sensor’s overall efficiency. A notable advantage of the DBIP sensor is its exceptional selectivity, capable of distinguishing the target bacteria from closely related bacterial strains, including different serotypes. Moreover, the sensor exhibits high sensitivity, showcasing a low detection limit of approximately 9 CFU mL^−1^. The sensor’s reusability further enhances its cost-effectiveness, reducing the need for frequent sensor replacements. The practicality of the DBIP sensor was demonstrated in the analysis of real apple juice samples, yielding good recoveries. The integration of quick fabrication, high selectivity, rapid response, sensitivity, and reusability makes the DBIP sensor a promising solution for monitoring pathogenic bacteria, playing a crucial role in ensuring food safety and safeguarding public health.

## 1. Introduction

In recent times, global food safety issues arising from pathogenic bacteria have sparked considerable concern due to a rise in severe food poisoning cases, particularly among vulnerable populations such as children, the elderly, and immunocompromised individuals [1,2]. Among the plethora of pathogenic bacteria, *Escherichia coli* O157:H7 (*E. coli* O157:H7) and *Staphylococcus aureus* (*S. aureus*) have gained notoriety for their role in causing severe foodborne illnesses and infections [3,4]. *E. coli* O157:H7 infection can lead to distressing symptoms, including abdominal cramps, bloody diarrhea, vomiting, and fever [5,6]. In more severe instances, it may progress to hemolytic uremic syndrome (HUS), which poses serious risks, particularly to young children and the elderly, often resulting in kidney failure and other complications [7]. On the other hand, *S. aureus* can cause a wide range of infections, from minor skin issues, such as boils and abscesses, to life-threatening conditions, such as pneumonia, bloodstream infections (bacteremia), and surgical site infections [8,9]. Of particular concern with *S. aureus* is its ability to develop resistance to multiple antibiotics, making effective treatment challenging [9]. Consequently, the development of an efficient and reliable strategy for detecting these pathogens is of utmost importance to safeguard food safety and public health.

Conventional culture-based methods have long been considered the gold standard for bacterial detection [10]. However, these methods often involve tedious procedures, including bacterial culturing steps and subsequent biochemical or serological tests, leading to time-consuming and labor-intensive processes [11]. While polymerase chain reaction (PCR) and enzyme-linked immunosorbent assay (ELISA) offer relatively rapid alternatives, they still require several hours to generate results [12,13,14,15,16]. Additionally, these methods are constrained by their high cost, complex operations, and reliance on prior knowledge of target sequences or the availability of specific antibodies/antigens [17]. Furthermore, their limited ability to distinguish closely related bacterial strains within a species poses challenges, as certain strains may share highly conserved DNA sequences, or antibodies/antigens may target common epitopes present across strains. As a consequence, there is a pressing demand for more rapid, user-friendly, cost-effective, and specific detection strategies for pathogenic bacteria.

Electrochemical sensors have emerged as promising tools for pathogen detection due to their rapid response, user-friendly nature, affordability, and potential for miniaturization [18]. Notably, these sensors offer a significant advantage by directly detecting whole bacterial cells without the need for time-consuming procedures such as cell lysis, nucleic acid extraction, or signal amplification. In the development of electrochemical sensors, the selection of appropriate receptors and their effective attachment to the transducer surface (e.g., glass carbon, gold, etc.) play a critical role. Various receptors, including antibodies, aptamers, phages, and carbohydrates, can be employed to target bacteria [19,20,21,22,23]. Among these, antibodies are the most commonly used recognition elements due to their exceptional selectivity and binding affinity. However, antibodies come with certain limitations, including their reliance on animal production, high expenses, and susceptibility to harsh conditions such as high temperatures, salt concentrations, strong acids or bases, and organic solvents [24,25]. Another challenge with using antibodies is the potential for denaturation or conformational changes when immobilized on the transducer surface through adsorption or covalent coupling [26].

Molecularly imprinted polymers (MIPs) are synthetic receptors that can be tailored to have precise binding sites that match a specific template, often representing the target analyte of interest [27,28]. Compared to antibodies, MIPs offer the benefits of simple preparation, cost-effectiveness, and enhanced physical and chemical stability, making them potential substitutes for natural antibodies [29,30]. While bacteria-imprinted polymers (BIPs) have shown promise in identifying single types of bacteria using a single bacteria template [31,32], real-life scenarios often involve co-contamination, with multiple species or strains of bacteria present simultaneously [33]. Therefore, it becomes essential to develop BIPs with multiple recognition sites capable of capturing multiple types of bacteria concurrently. However, the research on imprinting multiple-template bacteria remains limited, with only a few existing studies available [34,35]. Existing studies face certain limitations, such as lengthy preparation times for BIPs, often exceeding 48 h, and the necessity for additional measures, including drive dielectrophoretic or machine learning assistance. These prolonged preparation times can hinder the application of BIPs in situations requiring quick results or time-sensitive experiments. Additionally, the incorporation of extra measures can introduce complexity to the experimental setup and demand expertise in specific domains, thereby limiting their applicability in certain areas.

Building upon the preceding description, this study presents a novel approach for the highly selective screening of pathogenic bacteria by constructing a simple and robust electrochemical detection system based on a dual bacteria-imprinted polymer (DBIP) with double recognition sites. We demonstrate the efficacy of the DBIP sensor using two prominent pathogens, *E. coli* O157:H7 and *S. aureus*, as examples. The fabrication of the DBIP involves a facile and in situ electro-polymerization-based imprinting process, which results in the direct formation of highly specific binding sites tailored to the target bacteria on the electrode surface. The subsequent recognition and capture of the target bacteria induced detectable changes in the electrochemical impedance signal, enabling quantitative analysis. A key advantage of the electro-polymerization technique is its ability to control the deposition thickness, ensuring a thin layer of bacterial surface imprinting. This feature facilitates more rapid rebinding and unbinding of the target bacteria, leading to a faster recognition and regeneration process. By combining the unique advantages of MIPs with the inherent sensitivity and versatility of electrochemical techniques, this proposed sensor holds promise for highly selective, rapid, and sensitive detection of pathogens. The performance of the DBIP sensor was comprehensively evaluated, focusing on critical aspects such as selectivity, sensitivity, reusability, and practical applicability. Notably, we achieved a remarkable reduction in the preparation and recognition times for the DBIP, requiring only 20 and 10 min, respectively. The outcomes of this investigation hold the potential to advance the development of detection platforms for rapid and reliable identification of pathogenic bacteria, thereby enhancing food safety and public health surveillance.

## 2. Experimental Section

### 2.1. Materials and Reagents

The bacterial strains used in the experiment included *Staphylococcus aureus* (*S. aureus* ATCC 27661), *Escherichia coli* O157:H7 (*E. coli* O157:H7, ATCC 43889), *Escherichia coli* O6 (*E. coli* O6, ATCC 25922), and *Streptococcus hemolyticus* (*S. hemolyticus* ATCC 21059). LB liquid medium, trypsin soy broth, nutrient agar, Baird-Parker agar, egg-yolk tellurite emulsion, acetic acid (HAc), and potassium chloride (KCl) were purchased from Sangon Biotech (Shanghai, China). Lysozyme, cetyltrimethylammonium bromide (CTAB), and dimethyl sulfoxide (DMSO) were obtained from BBI Life Sciences Corporation (Shanghai, China). *o*-phenylenediamine (*o*PD) was obtained from TCI (Shanghai, China). Milli-Q grade (>18 MΩ) water was used throughout the experiment.

### 2.2. Apparatus and Measurements

All electrochemical experiments were conducted using a CHI 660E workstation with a standard three-electrode system. The glass carbon electrode (GCE, 3 mm in diameter), platinum sheet, and saturated calomel electrode (SCE) serve as the working, auxiliary, and reference electrodes, respectively. The cyclic voltammetry (CV) measurements were recorded in 0.1 M KCl solution containing 5 mM K_3_[Fe(CN)_6_]. The electrochemical impedance spectroscopy (EIS) was performed in 0.1 M KCl solution containing 1 mM K_3_[Fe(CN)_6_] and 1 mM K_4_[Fe(CN)_6_] by applying an open circuit voltage over a frequency range of 0.1 to 100,000 Hz with an amplitude of 5 mV.

### 2.3. Bacterial Cultivation

*E. coli* O157:H7 and *E. coli* O6 were cultured individually in LB liquid medium at 37 ℃ overnight with continuous agitation at 200 rpm. Similarly, *S. aureus* and *S. hemolyticus* were cultured separately in trypsin soy broth medium at 37 °C overnight under continuous agitation at 200 rpm. Enumeration of bacterial colonies was performed using the plate count technique. For subsequent experiments, the bacteria were rendered nonviable by treating the cultures with formaldehyde at a 1:100 ratio. Throughout the entirety of the experimental procedures, the formaldehyde-treated inactivated bacterial cultures were utilized. Subsequently, the bacterial cultures were centrifuged at 10,000 rpm for 3 min to pellet the bacterial cells, which were then subjected to two rounds of washing to remove the residual culture medium. Following the removal of the culture medium, the bacterial pellet was resuspended in a specific volume of 0.01 M sterile phosphate-buffered solution at pH 7.4. This resuspended bacterial suspension underwent a series of 10-fold serial dilutions to generate a range of dilutions with varying concentration gradients, ranging from 10 to 10^6^ CFU mL^−1^. These prepared dilutions were subsequently employed in the experimental protocols.

### 2.4. Preparation of the DBIP-Modified Electrode

Before use, the GCE was polished with 0.3−0.05 μm of alumina aqueous slurry until it had a shiny appearance. Then, the polished GCE was immersed in acetate buffer solution (0.1 M, pH 5.8) containing *o*PD (5 mM) and double bacterial template of *E. coli* O157:H7 and *S. aureus* (both at 10^8^ CFU mL^−1^), and CV was carried out under gentle stirring for 15 cycles with a potential range of −0.05 to 0.95 V vs. SCE and a scan rate of 0.05 V s^−1^ [36]. To elute the bacterial template, the modified electrode was soaked in CTAB/HAc solution (1 mM CTAB dispersed in 36% HAc) at 37 °C for 10 min under constant shaking (400 rpm). The fabricated modified electrodes before and after template removal were named P*o*PD+dual bacteria/GCE and DBIP/GCE, respectively. A non-imprinted polymer (NIP)-modified electrode (NIP/GCE) was prepared using the same steps but without adding the bacterial template.

### 2.5. Detection of E. coli O157:H7 and S. aureus

For capture, the freshly prepared DBIP/GCE was incubated with 250 μL of phosphate-buffered solution (0.01 M, pH 7.4) containing a specific concentration of bacteria at 37 °C for 15 min under constant shaking (300 rpm). The fabricated modified electrode was denoted as DBIP-(*E. coli* O157:H7+*S. aureus*)/GCE. Following this, the DBIP-(*E. coli* O157:H7+*S. aureus*)/GCE was washed with deionized water and analyzed by EIS under the conditions mentioned above.

### 2.6. Optimization of Experimental Conditions

To obtain better sensing performances, several parameters involved in the DBIP preparation (concentration of monomer and bacterial template, polymerization cycles, and conditions for template elution) and bacterial recognition (time, pH, and oscillation speed) were systematically optimized. The selection of optimum eluents and elution time for template removal was guided by the degree of reduction in charge transfer resistance (*R*_ct_). A greater reduction in impedance indicated more effective template removal. Other optimal conditions were chosen based on the EIS response (Δ*R*/*R*) towards a mixture of *E. coli* O157:H7 and *S. aureus*, each at a concentration of 10^5^ CFU mL^−1^. The Δ*R*/*R* was calculated using the following formula:

Δ*R*/*R* = (*R*_cta_ − *R*_ctb_)/*R*_ctb_

Here, *R*_ctb_ and *R*_cta_ represent the values of *R*_ct_ before and after capturing the target bacterial template, respectively.

### 2.7. Real Sample

To assess the sensor’s suitability for real-world applications, we selected a sample of apple juice purchased from a local supermarket as our test specimen. We diluted the apple juice 100 times and then introduced different amounts of *E. coli* O157:H7 or *S. aureus* into separate portions, achieving final concentrations ranging from 10^2^ to 10^4^ CFU mL^−1^. As a control, we conducted additional experiments using a phosphate-buffered solution (0.01 M, pH 7.4) instead of the bacteria. Subsequent incubation with the DBIP/GCE and EIS detection followed the same procedures used for the pure bacterial solution. Each group underwent at least three parallel experiments to ensure reliable and consistent results.

## 3. Results and Discussion

### 3.1. Fabrication of DBIP-Modified Electrode for Bacterial Capture and Detection

**Scheme 1:**
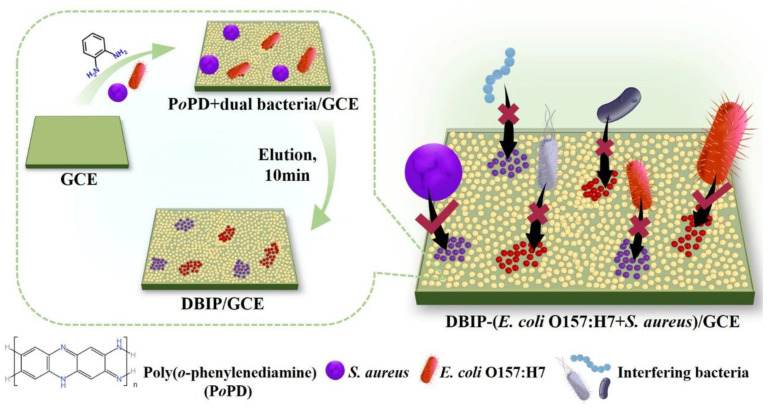
Preparation of dual bacteria-imprinted polymer (DBIP) for simultaneous capture of *E. coli* O157:H7 and *S. aureus*.

Scheme 1 illustrates the preparation of the dual-template bacteria-imprinted polymer (DBIP) designed to simultaneously capture *E. coli* O157:H7 and *S. aureus*. The DBIP was efficiently fabricated on a GCE surface using cyclic voltammetry electro-copolymerization of *o*-phenylenediamine (*o*PD) monomer along with dual bacterial templates (*E. coli* O157:H7 and *S. aureus*). Subsequently, in-situ elution of the templates was performed for 10 min. The entire preparation process of the DBIP sensor is remarkably swift, taking just 20 min, which is much faster than the majority of reported electrochemical sensors designed for pathogen detection.

The resulting DBIP on the electrode surface exhibits rapid and selective recognition of the target bacteria from complex bacterial mixtures in a mere 15 min. This specificity arises from the formation of imprinted sites within the polymer, which are tailored to complement the size, shape, and chemical characteristics of the target bacteria. Considering the low conductivity of bacteria, the target *E. coli* O157:H7 or *S. aureus* captured by the DBIP can be individually analyzed using the EIS technique.

The developed DBIP-modified electrode provides a promising platform for the capture and detection of specific bacteria, paving the way for potential applications in various fields, including food safety monitoring and biomedical diagnostics.

### 3.2. Characterization of the DBIP Fabrication

**Figure 1:**
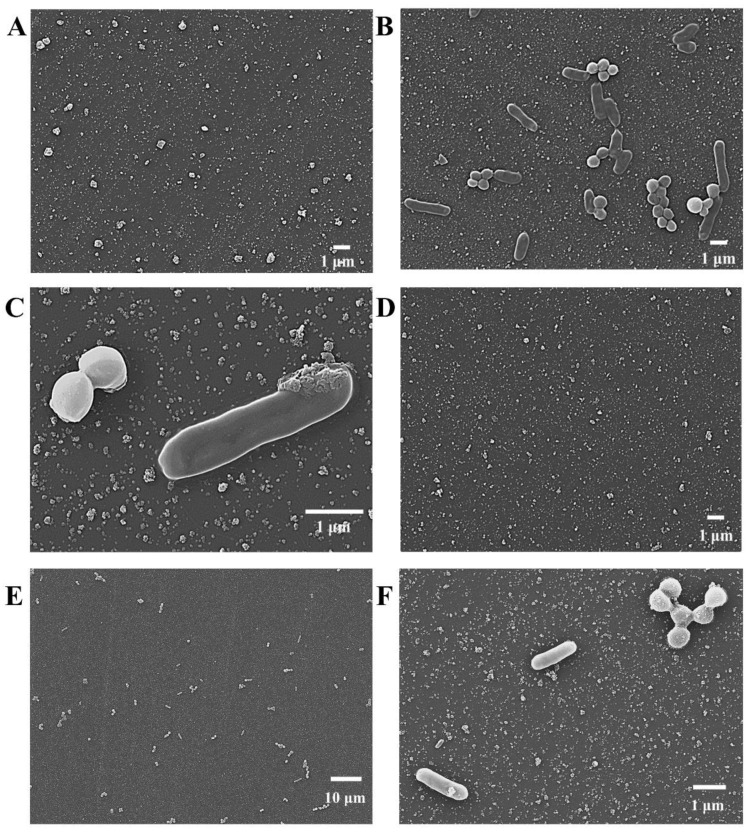
SEM images showing the morphological changes in the electrode surface at various stages during the preparation of the DBIP. (**A**) P*o*PD/GCE, (**B**,**C**) P*o*PD+dual bacteria/GCE with 5000- and 20,000-fold magnification, (**D**) DBIP/GCE, (**E**,**F**) DBIP-(*E. coli* O157:H7+*S. aureus*)/GCE with 1000- and 10,000-fold magnification.

The morphological changes of the electrode surface during the fabrication of the DBIP were investigated using scanning electron microscopy (SEM). In the absence of bacteria, during the electro-polymerization of *o*PD, only a few irregular aggregates were observed on the electrode surface (Figure 1A). However, in the presence of the dual bacterial template (*E. coli* O157:H7 and *S. aureus*), numerous well-defined rod-shaped and globule-shaped bacteria were observed to be embedded within the polymer matrix on the electrode surface (Figure 1B). Further magnification provided a clearer view of the two distinct types of bacteria, with the rod-shaped bacteria measuring approximately 2 μm in length and 0.6 μm in width, and the globule-shaped bacteria having a diameter of about 0.8 μm (Figure 1C). These dimensions align well with the reported sizes of *E. coli* O157:H7 and *S. aureus*, respectively [32,37]. The presence of the dual bacterial template within the P*o*PD matrices was confirmed by these results. However, after eluting the copolymerized film for 10 min, almost no bacteria were found on the electrode surface (Figure 1D). This observation indicates that the bacteria were successfully removed from the P*o*PD matrices during the elution process. The DBIP, featuring a cavity-free structural trait, provides enhanced accessibility, thereby enabling the rapid recognition of the target bacteria [38]. Following the recognition of the dual target bacteria, namely *E. coli* O157:H7 and *S. aureus*, within a rapid timeframe of 15 min (Figure 1E), a subsequent observation revealed the reappearance of numerous rod-shaped and globule-shaped bacteria reappearing on the electrode surface. From the further magnified image (Figure 1F), the distinct morphologies of the two bacterial types become more apparent.

### 3.3. Electrochemical Characterization of the DBIP-Based Sensor

**Figure 2:**
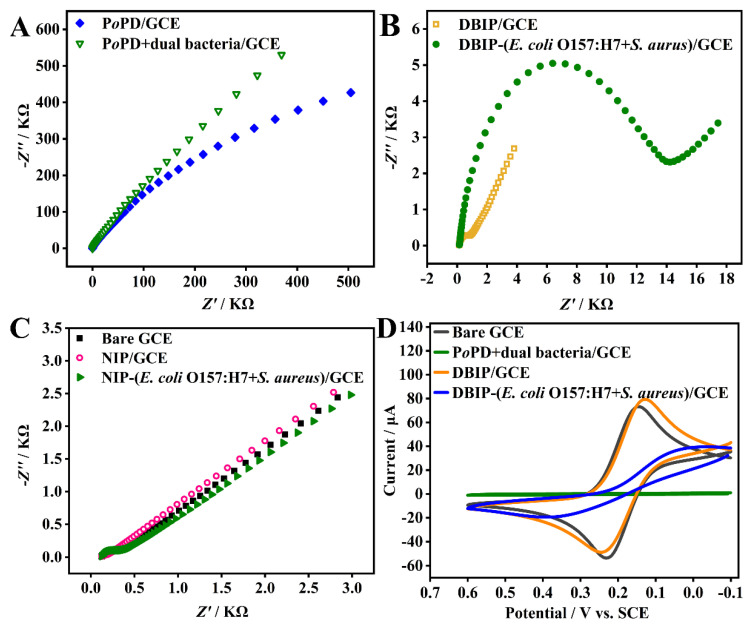
Electrochemical characterization of the DBIP fabrication and recognition. (**A**–**C**) EIS Nyquist plots and (**D**) CV curves of different modified electrodes as indicated.

The DBIP fabrication processes and the recognition response were thoroughly characterized using EIS and CV. Figure 2A–C represent the EIS Nyquist plots of different modified GCEs. The bare GCE displayed an almost straight line (Figure 2C, solid square), indicating a mass diffusion-limited electron transfer process [39]. The impedance behavior was high when the dual bacterial template was electro-polymerized onto the GCE surface with *o*PD monomer, owing to the non-conductive nature of both bacteria and P*o*PD. However, after elution with CTAB/HAc for 10 min, the impedance substantially decreased to around 400 Ω for the modified electrode (DBIP/GCE) (Figure 2B, hollow square), confirming the successful removal of the bacterial template.

The fabricated DBIP, acting as the recognition element, efficiently captured the target *E. coli* O157:H7 and *S. aureus*, each at a concentration of 10^5^ CFU mL^−1^. The semicircular diameter of the modified electrode (Figure 2B, solid circle) was much larger than that of the DBIP. Conversely, the non-imprinted polymer (NIP) showed almost no recognition response toward the template bacteria (Figure 2C). These results indicate that the DBIP can specifically recognize the target bacteria due to the imprinted sites formed on the polymer matrices with complementary physical and chemical characteristics to the template bacteria.

These findings were further corroborated by the CV analysis shown in Figure 2D. The bare GCE showed a pair of distinct redox peaks attributed to the redox behavior of the electroactive ion pair [Fe(CN)_6_] ^3−/4−^. However, after the electro-copolymerization of *o*PD and the dual bacterial template, the redox peak disappeared. Upon elution, the fabricated DBIP exhibited an evident redox peak, confirming the successful removal of the dual bacterial template from the P*o*PD matrices. Subsequently, when the DBIP/GCE was exposed to a phosphate-buffered solution (0.01 M, pH 7.4) containing the dual template bacteria, the redox peak current decreased, and the Δ*E*_p_ (potential separation between the cathodic and anodic peaks) increased significantly. These changes indicated that the target bacteria were captured by the DBIP, leading to hindered electron transfer of [Fe(CN)_6_] ^3−/4−^ over the GCE surface.

In summary, the electrochemical characterization using EIS and CV demonstrates the successful fabrication of the DBIP sensor and its efficient recognition of the target bacteria through specific imprinted sites, showcasing its potential for selective bacterial capture and detection applications.

### 3.4. Optimization of Experimental Conditions

**Figure 3:**
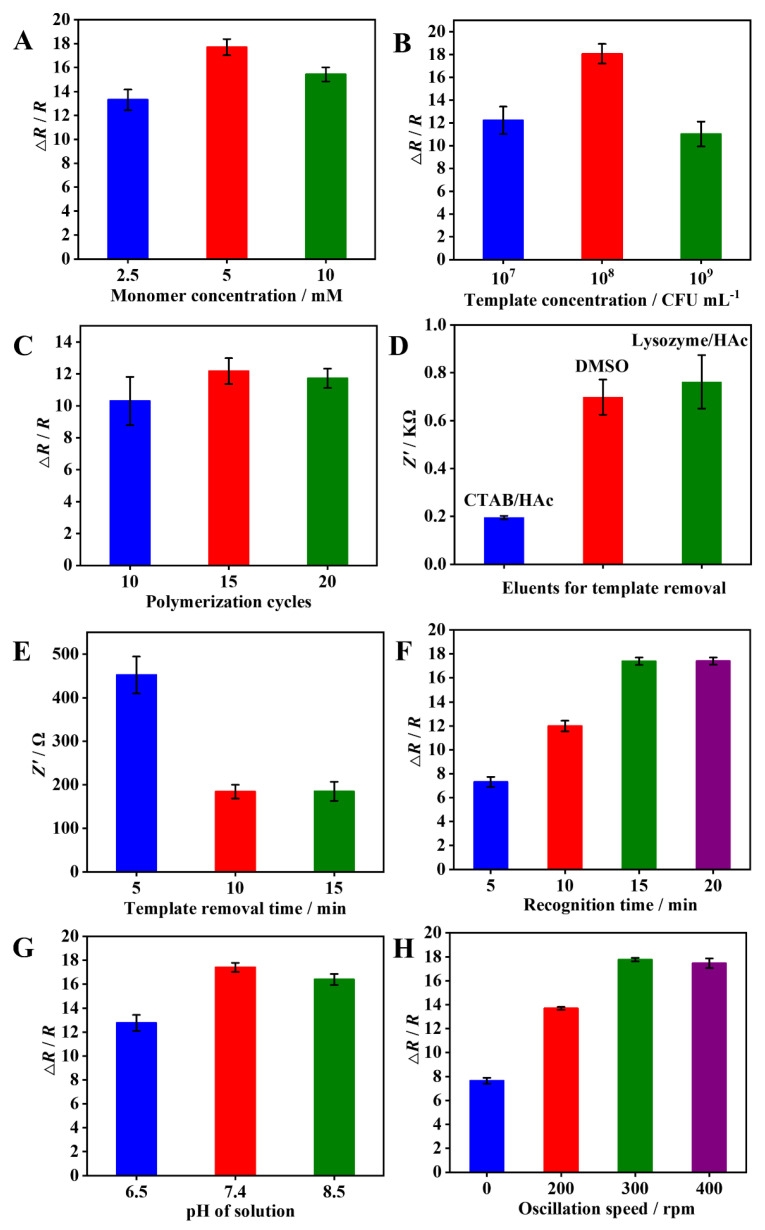
Optimization of parameters for the DBIP fabrication and bacterial recognition: (**A**–**B**) concentration of monomer and bacterial template during electro-polymerization; (**C**) number of polymerization cycles; (**D**–**E**) different eluents and elution time for removal of bacterial template; (**F**–**H**) recognition time, pH condition, and oscillation speed during the recognition process.

The achievement of optimal sensing performance necessitates the optimization of various parameters related to the fabrication and recognition processes of the DBIP. The first step involved the optimization of electro-polymerization conditions, specifically focusing on the concentrations of *o*PD monomer and the dual bacterial template, as well as the number of polymerization cycles. Higher concentrations of *o*PD or the bacterial template led to an increase in the EIS response up to a certain point, beyond which the response declined. After careful evaluation (Figure 3A,B), the optimal concentrations of *o*PD and the bacterial template were determined to be 5 mM and 10^8^ CFU mL^−1^, respectively. The polymerization cycle was also critical, affecting the thickness of the imprinted layer and the generation of sufficient imprinted sites. After extensive testing (Figure 3C), 15 cycles of polymerization demonstrated the highest recognition response.

The second step focused on optimizing template elution conditions, including the choice of eluents and elution time. Comparative assessment of three eluents, namely DMSO, lysozyme (10 mg mL^−1^), and CTAB/HAc, revealed CTAB/HAc to be the most effective eluent (Figure 3D). Subsequently, the elution time with CTAB/HAc was fine-tuned, with 10 min being the optimal duration for the successful removal of the dual bacterial template (Figure 3E).

To further enhance the sensing performance, the recognition conditions were refined, encompassing recognition time, pH, and oscillation speed. Extensive investigations (Figure 3F–H) identified the optimal recognition time as 15 min, the preferred pH value as 7.4, and the optimal oscillation speed as 300 rpm.

### 3.5. Quantitative Detection of E. coli O157:H7 and S. aureus

Under the optimized experimental conditions, we conducted separate quantitative detection of *E. coli* O157:H7 and *S. aureus* using the DBIP-based sensor. To achieve this, 10-fold serially diluted samples of *E. coli* O157:H7 and *S. aureus* (ranging from 10^1^ to 10^6^ CFU mL^−1^) were individually incubated with the DBIP/GCE for 15 min. The subsequent quantitative detection was performed using the EIS strategy. Each sample was subjected to at least three parallel experiments to ensure reliability.

**Figure 4:**
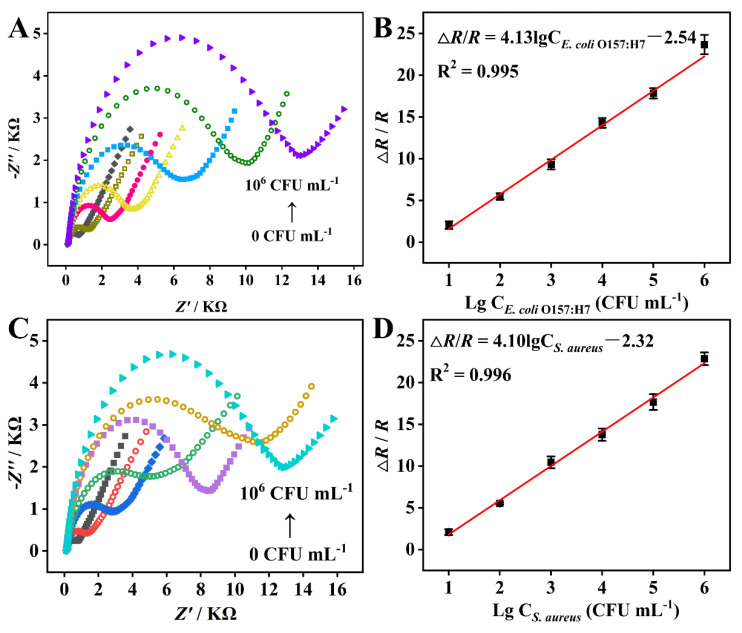
(**A**,**C**) EIS Nyquist plots and (**B**,**D**) corresponding calibration curves obtained from the DBIP sensor for the separate quantitative detection of *E. coli* O157:H7 and *S. aureus* over a range of concentrations from 0 to 10^6^ CFU mL^−1^.

Figure 4A,C display the EIS Nyquist plots of the DBIP-based sensor for the separate detection of *E. coli* O157:H7 and *S. aureus*. As the bacterial concentration increased from 0 to 10^6^ CFU mL^−1^, the impedances also increased. This behavior can be attributed to the partial blocking of electron transfer between the redox probe and the electrode surface caused by the captured bacterial cells on the DBIP/GCE, leading to an increase in film impedance [31]. Figure 4B,D present the corresponding calibration curves of the EIS response plotted against the logarithmic concentration of *E. coli* O157:H7 and *S. aureus*, respectively. Over a wide concentration range from 10 to 10^6^ CFU mL^−1^, both calibration curves demonstrated excellent linearity between ΔR/R (Ω) and the logarithmic concentration of *E. coli* O157:H7 or *S. aureus*. The linear regression equations were expressed as Δ*R/R* (Ω) = 4.13 lg *C_E.coli_*_O157:H7_−2.54 (R^2^ = 0.995), and Δ*R/R* (Ω) = 4.10 lg *C_S. aureus_*−2.32 (R^2^ = 0.996), respectively. Based on the 3σ/S rule [17], the calculated detection limits for *E. coli* O157:H7 and *S. aureus* were found to be 9.4 and 9.5 CFU mL^−1^, respectively. These results indicate that the DBIP-based sensor exhibits remarkable sensitivity in quantitatively detecting both *E. coli* O157:H7 and *S. aureus* over a broad concentration range, making it a promising tool for rapid bacterial detection in various applications.

### 3.6. Selectivity of the DBIP Sensor

**Figure 5:**
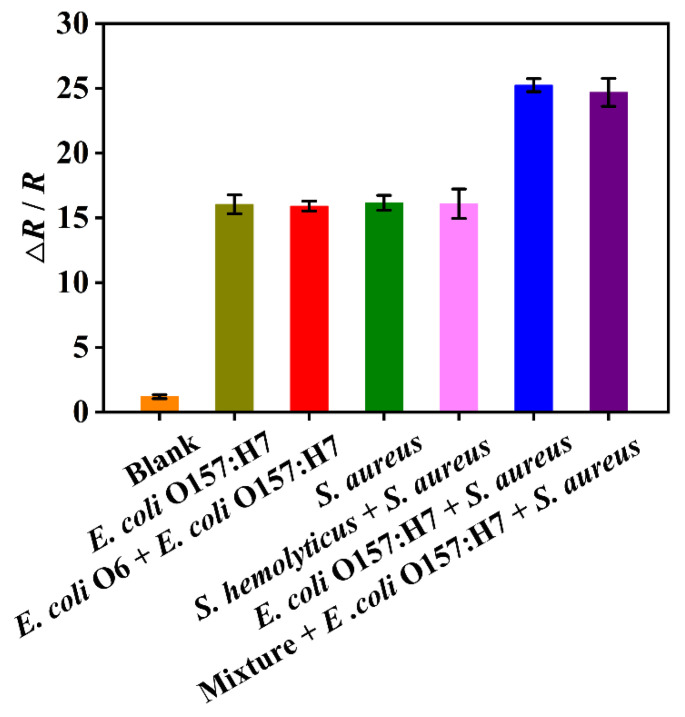
Selectivity of the DBIP sensor for detection of *E. coli* O157:H7 and *S. aureus*. Mixture refers to a mixed bacterial solution of *E. coli* O6 and *S. hemolyticus*. Each bacterium in the different samples was kept at the same concentration of 10^5^ CFU mL^−1^.

The sensor’s selectivity for bacteria is a crucial factor that enhances its potential applicability in diverse fields, including food safety, environmental monitoring, and clinical diagnostics. To assess the selectivity of the DBIP with dual recognition sites, we chose *E. coli* O6 and *S. hemolyticus* as potential interferences. These strains were selected due to their close resemblance to *E. coli* O157:H7 and *S. aureus*, respectively. As shown in Figure 5, the EIS response for single *E. coli* O157:H7 and *S. aureus* was significantly higher than that for the blank phosphate-buffered solution (0.01 M, pH 7.4), indicating clear and distinct detection of these target bacteria. Remarkably, the response to dual bacteria (both *E. coli* O157:H7 and *S. aureus*) was even more pronounced than that of the single bacterium, demonstrating an enhanced recognition response when detecting both strains simultaneously. Notably, the EIS recognition response to either single *E. coli* O157:H7 and *S. aureus*, or the dual bacteria, remained unaffected by the presence of closely related strains, such as *E. coli* O6 and/or *S. hemolyticus*. These findings demonstrate the outstanding selectivity of the DBIP sensor.

### 3.7. Reusability of the DBIP-Based Sensor

**Figure 6:**
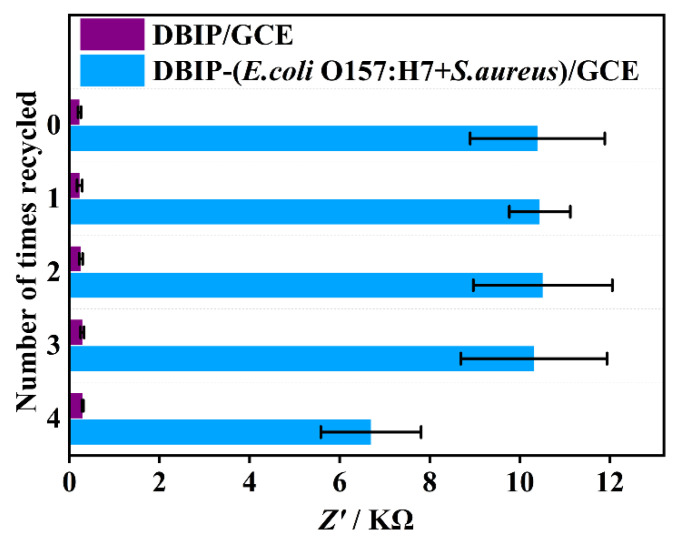
Reusability of the DBIP sensor for bacteria detection.

The DBIP-based sensing interface exhibited efficient regeneration capability within a short duration of 15 min, achieved by removing the rebound bacteria using CTAB/HAc, as previously mentioned. Figure 6 illustrates the successful and clean removal of rebound bacteria, and remarkably, after being recycled four times, the sensor’s response signal remained at 64.4% of its initial value. This finding reveals the excellent reusability of the DBIP-based sensor, significantly reducing the need for frequent sensor replacements or replenishments. As a result, this reusability feature offers notable advantages in terms of cost and time savings. The sensor’s ability to maintain its detection performance even after multiple recycling cycles enhances its practicality and sustainability, making it a promising choice for various applications in fields such as food safety, environmental monitoring, and clinical diagnostics.

### 3.8. Detection of E. coli O157:H7 and S. aureus in Real Samples

To validate the practical applicability of the DBIP-based sensor in detecting *E. coli* O157:H7 and *S. aureus* in real samples, we utilized apple juice samples as a representative example. The obtained results are summarized in Table 1. The recovery rates for *E. coli* O157:H7 and *S. aureus* ranged from 86.86% to 98.40% and 81.36% to 100.58%, respectively. These findings demonstrate the sensor’s effectiveness in detecting the target bacteria in actual samples. The high recovery rates highlight the sensor’s reliability and accuracy in real-world applications.

## 4. Conclusions

In summary, we have successfully developed a simple, and reusable impedimetric sensor based on a novel dual bacteria-imprinted polymer for highly selective, rapid, and sensitive detection of pathogenic bacteria. The remarkable fabrication time of just 20 min renders the DBIP sensor an expedient and practical tool for real-world applications. The outstanding selectivity exhibited by the DBIP sensor in distinguishing between bacterial serotypes and closely related strains can be attributed to the formation of complementary binding sites that precisely match the unique features of the target bacteria. The rapid recognition and regeneration process of the DBIP sensor is made possible by the presence of cavity-free imprinted sites on its surface. This unique structural feature enables enhanced mass transfer and binding kinetics, resulting in faster and more efficient binding and unbinding of the target bacteria. The impressive combination of quick fabrication, superior selectivity, rapid response, sensitivity, and reusability underscores the great potential of DBIP-based sensors as versatile and cost-effective solutions for monitoring pathogenic bacteria across diverse fields such as food safety, environmental monitoring, and clinical diagnostics. Nevertheless, the DBIP sensor does exhibit certain limitations. Notably, it currently lacks the capability to conduct simultaneous quantitative analyses of two types of bacteria.

## Figures and Tables

**Table 1:** Detection of *E. coli* O157:H7 and *S. aureus* in apple juice (*n* = 3).

Sample	Bacteria	Original (CFU mL^−1^)	Added (CFU mL^−1^)	Found (Mean ± SD/CFU mL^−1^)	Recovery (%)
Apple juice	*E. coli* O157:H7	Not found	10^2^	(9.84 ± 0.82) × 10^1^	98.4
			10^3^	(9.80 ± 1.35) × 10^2^	98.0
			10^4^	(8.69 ± 1.16) × 10^3^	86.9
	*S. aureus*		10^2^	(9.36 ± 0.98) × 10^1^	93.6
			10^3^	(8.14 ± 0.80) × 10^2^	81.4
			10^4^	(1.01 ± 0.02) × 10^4^	101
